# Protrusion of nasobiliary drainage catheter through the anus

**DOI:** 10.1093/gastro/goag086

**Published:** 2026-09-08

**Authors:** Dongrui Li, Yuanbo Bi, Shengxiong Chen, Tengfei Zhang, Yunfei Liang, Jiansheng Zhang

**Affiliations:** Department of Hepatobiliary and Pancreatic Surgery, The Second Hospital of Hebei Medical University, Shijiazhuang, Hebei, 050000, P. R. China; Department of Hepatobiliary and Pancreatic Surgery, The Second Hospital of Hebei Medical University, Shijiazhuang, Hebei, 050000, P. R. China; Department of Hepatobiliary and Pancreatic Surgery, The Second Hospital of Hebei Medical University, Shijiazhuang, Hebei, 050000, P. R. China; Department of Hepatobiliary and Pancreatic Surgery, The Second Hospital of Hebei Medical University, Shijiazhuang, Hebei, 050000, P. R. China; Department of Hepatobiliary and Pancreatic Surgery, The Second Hospital of Hebei Medical University, Shijiazhuang, Hebei, 050000, P. R. China; Department of Hepatobiliary and Pancreatic Surgery, The Second Hospital of Hebei Medical University, Shijiazhuang, Hebei, 050000, P. R. China

## Introduction

Choledocholithiasis, the presence of stones in the common bile duct, is the most frequent cause of cholangitis, which is inflammation and infection of this duct. This inflammatory process arises from bile backing up due to the obstruction caused by the gall stones. It can progress to severe acute cholangitis with a high mortality rate, and prompt decompression is required [1–3]. Endoscopic nasobiliary drainage (ENBD) is a critical component in the management of both acute cholangitis and obstructive jaundice [4]. By providing external drainage of bile, ENBD promptly decompresses the obstructed biliary tree [5]. A randomized controlled trial has demonstrated that performing ENBD after endoscopic sphincterotomy can significantly reduce the incidence of postoperative pancreatitis and cholangitis [6].

In clinical practice, a significant concern regarding ENBD is the potential for nasobiliary catheter migration into the intestinal tract [7]. While biliary stent displacement is widely documented, reports on ENBD catheter migration are still scarce, typically restricted to dislodgement from the duodenal papilla or exiting through the oral cavity [8–10]. Herein, we reported a case of spontaneous nasobiliary catheter expulsion via the anal canal following complete dislodgement.

## Case report

We reported an exceptionally rare case of a 55-year-old woman (160 cm, 73 kg) who was initially diagnosed with choledocholithiasis and cholelithiasis at a local hospital. Repeat contrast-enhanced abdominal computed tomography at our center confirmed common bile duct (CBD) stones, and subsequent magnetic-resonance cholangiopancreatography revealed acute cholecystitis, multiple gall-bladder stones, an impacted stone at the distal CBD, CBD dilatation and biliary sludge. Endoscopic retrograde cholangiopancreatography (ERCP) was performed the following day. Balloon dilation of the pancreatic duct was undertaken and a 250-cm ENBD catheter (Mro-tech, Nanjing, China) was inserted. The surgical procedure went smoothly, and the catheter was placed correctly with the tail end being pigtail (Supplementary Material 1). Prepare medical adhesive tape and cut it into a “Y” shape. The center point of the “Y” shape adhesive tape is tightly adhered to the same side of the nasal ala. The two branches are wound around the outside of the nasobiliary tube and fixed to the adhesive tape on the nasal ala. The bile drainage after the operation was good.

**Figure 1:**
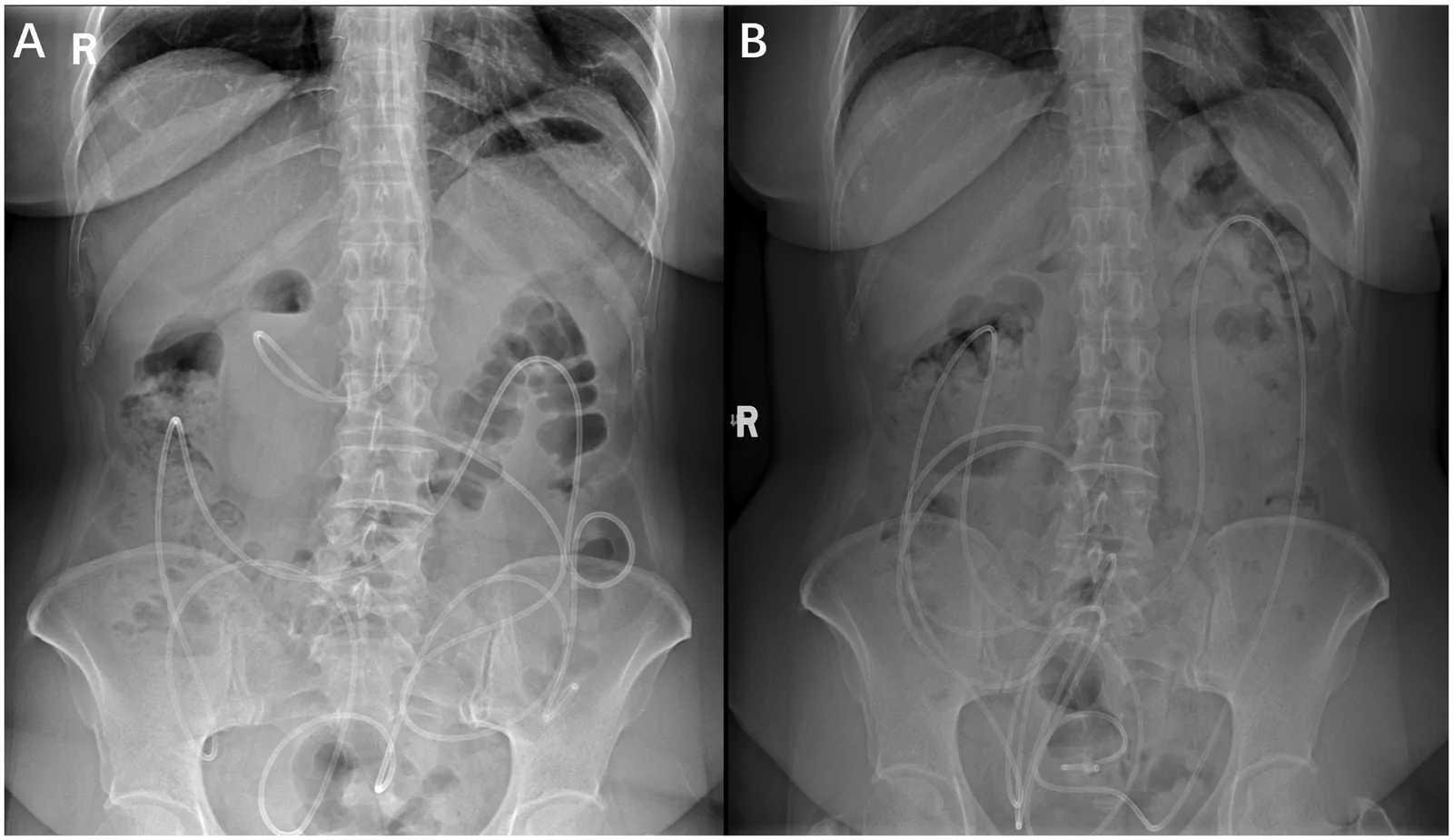
(A) Radiography revealed a tubular shadow in the region of the esophagus, stomach, and intestines, with the distal end located within the intestinal loops in the left lower abdomen. (B) Radiography showed a tubular shadow in the abdominal intestinal region, with the proximal end located within the pelvic cavity.

On post-ERCP day (PED) 3, the external portion of the ENBD catheter was noted to have shortened; an erect abdominal radiograph was therefore obtained to locate its distal end. The film demonstrated a tubular radio-opacity following the course of the esophagus, stomach and bowel, with the tip projected over the left-lower quadrant (Fig. 1A). On PED 4 the proximal end of the catheter was found protruding from the anus (Supplementary Material 2). Repeat radiography showed the catheter lying within the intestinal lumen, its tip in the pelvic cavity (Fig. 1B). Complete trans-anal expulsion of the ENBD catheter occurred on PED 9 and the patient reported no discomfort. Laparoscopic cholecystectomy was successfully performed on PED 13 (Supplementary Material 3).

## Discussion

We reported an extremely rare case in which a nasobiliary drain, placed during ERCP, was expelled from the anus on PED 3. Remarkably, the 250-cm catheter had traversed the entire gastrointestinal tract and its distal end was located in the sigmoid colon, yet the patient remained asymptomatic and computed tomography revealed no evidence of intestinal perforation or fistula.

As no comparable cases have been documented previously, we analyzed the etiology of this ENBD catheter dislodgement. We hypothesized that three mechanisms were involved: suboptimal catheter placement during the procedure, external traction, sweat-related factors leading to loose external fixation, and displacement caused by postoperative gastrointestinal peristalsis. The traversing of the entire digestive tract by the catheter is tentatively ascribed to the inherent flexibility of the gastrointestinal tract.

There are few studies on the displacement of nasobiliary catheters. Gong’s research suggests that the external length of the nasobiliary catheter is a factor influencing displacement [10]. Their findings demonstrated that when the extracorporeal portion of the nasobiliary catheter measured less than 150 cm, the incidence of migration was approximately 10%. However, when the extracorporeal catheter length exceeded 150 cm, particularly within the 160–170 cm range, the migration incidence decreased significantly.

Given that standardized intraoperative protocols were strictly followed and the patient reported no accidental self-traction of the tube, we suggest that *in vivo* factors also play an important role in catheter displacement. Consequently, we hypothesize that the length of the catheter distributed across different gastrointestinal segments, along with the corresponding luminal anatomy, are key determinants of migration. Additionally, the patient’s peristaltic intensity and swallowing activity may also contribute significantly. To date, these correlations have not been clinically validated, offering a potential direction for future research. To avoid the repetition of such incidents, we advocate for the routine monitoring of the catheter’s external length, both after the procedure and during subsequent nursing maintenance.

A limitation of this case is that, because the patient reported no discomfort and imaging showed no signs of gastrointestinal perforation, follow-up endoscopic examinations (such as colonoscopy or upper endoscopy) were not performed to assess the presence of mucosal pressure ulcers or potential injuries.

## Conclusion

We reported a rare case of an ENBD catheter traversing the entire digestive tract and prolapsing through the anus. Given the limited clinical data currently available on ENBD catheter migration, this case underscores the necessity for further research into the factors contributing to such displacement. Furthermore, we recommend the routine measurement of the catheter’s external length, both postoperatively and during daily nursing care, to prevent the recurrence of such adverse events.

## Supplementary Material

goag086_Supplementary_Data

## Data Availability

No datasets were generated or analyzed in this study; therefore, data sharing is not applicable. Written informed consent was obtained from the patient.
